# Expression of an osmotin-like protein from *Solanum nigrum* confers drought tolerance in transgenic soybean

**DOI:** 10.1186/s12870-014-0343-y

**Published:** 2014-12-10

**Authors:** Ricardo Luís Mayer Weber, Beatriz Wiebke-Strohm, Christian Bredemeier, Márcia Margis-Pinheiro, Giovani Greigh de Brito, Ciliana Rechenmacher, Paulo Fernando Bertagnolli, Maria Eugênia Lisei de Sá, Magnólia de Araújo Campos, Regina Maria Santos de Amorim, Magda Aparecida Beneventi, Rogério Margis, Maria Fátima Grossi-de-Sa, Maria Helena Bodanese-Zanettini

**Affiliations:** Universidade Federal do Rio Grande do Sul, Porto Alegre, RS 91501-970 Brazil; Embrapa Clima Temperado, Pelotas, RS 96010-971 Brazil; Embrapa Trigo, Passo Fundo, 99001-970 RS Brazil; Empresa de Pesquisa Agropecuária de Minas Gerais, Uberaba, MG 38001-970 Brazil; Embrapa Recursos Genéticos e Biotecnologia, Brasilia, DF 70770-917 Brazil; Universidade Federal de Campina Grande, Cuité, PB 58175-000 Brazil; Departamento de Genética, Instituto de Biociências, Av, Bento Gonçalves, 9500, CP 15053, 91501-970 Porto Alegre, RS Brazil

**Keywords:** Abiotic stress, Bombardment, Drought tolerance, Genetic transformation, *Glycine max*, Osmotin, Water deficit

## Abstract

**Background:**

Drought is by far the most important environmental factor contributing to yield losses in crops, including soybeans [*Glycine max* (L.) Merr.]. To address this problem, a gene that encodes an osmotin-like protein isolated from *Solanum nigrum var. americanum* (*Sn*OLP) driven by the UBQ3 promoter from *Arabidopsis thaliana* was transferred into the soybean genome by particle bombardment.

**Results:**

Two independently transformed soybean lines expressing *Sn*OLP were produced. Segregation analyses indicated single-locus insertions for both lines. qPCR analysis suggested a single insertion of *SnOLP* in the genomes of both transgenic lines, but one copy of the *hpt* gene was inserted in the first line and two in the second line. Transgenic plants exhibited no remarkable phenotypic alterations in the seven analyzed generations. When subjected to water deficit, transgenic plants performed better than the control ones. Leaf physiological measurements revealed that transgenic soybean plants maintained higher leaf water potential at predawn, higher net CO_2_ assimilation rate, higher stomatal conductance and higher transpiration rate than non-transgenic plants. Grain production and 100-grain weight were affected by water supply. Decrease in grain productivity and 100-grain weight were observed for both transgenic and non-transgenic plants under water deficit; however, it was more pronounced for non-transgenic plants. Moreover, transgenic lines showed significantly higher 100-grain weight than non-transgenic plants under water shortage.

**Conclusions:**

This is the first report showing that expression of *Sn*OLP in transgenic soybeans improved physiological responses and yield components of plants when subjected to water deficit, highlighting the potential of this gene for biotechnological applications.

## Background

Soybean is one of the most important commodities in the world, and drought is one of the most relevant environmental factors contributing to yield losses. Long-term drought stress is a major problem, but in the short term, it also decreases crop production even when other conditions are favorable and may have serious economic and social consequences. In Brazil, the occurrence of prolonged drought during the soybean growing season has become increasingly common in recent years. This situation may become even more dramatic in light of current environmental change predictions, which point to global warming and the consequent occurrence of drought stress [1].

Molecular biology tools for the identification and transfer of genes responsible for drought tolerance have been applied to combat drought in soybean [1]. Different types of genetically modified plants have been developed to overcome water deficit, including the over expression of protective proteins.

Osmotins (osmotin-like proteins or OLPs) are members of the Pathogenesis-related protein 5 (PR-5) family [2], which are produced in plants under different abiotic and biotic stresses [3-6]. These proteins were identified in several plant species, including strawberry, *Fragaria ananassa* [6], *Arabidopsis thaliana* [7] and soybean, *Glycine max* [8,9].

Numerous studies have been performed to determine the physiological role of osmotin in stress tolerance, but the mechanism of its action remains unclear. It has been reported that tobacco osmotin overexpression in different plant species confers tolerance to abiotic stresses, especially salinity and drought. This response was observed in transgenic plants of tobacco, *Nicotiana tabacum* [10], wheat, *Triticum aestivum* [11], cotton, *Gossypium hirsutum* [12], tomato, *Solanum lycopersicum* [13], and soybean [14]. Tobacco osmotin can act in osmotolerance by facilitating the compartmentalization of solutes [10] or metabolic/structural alterations during osmotic adjustment [3,4].

The genus *Solanum* has a broad set of genes, including the PR-5 protein family, with the potential to confer resistance to pathogens [4]. Two PR5-like genes were isolated from the genome of black nightshade (*Solanum nigrum* L. var. *americanum*), a solanaceous weed. The predicted products of both genes, named *Sn*OLP (neutral) and *Sn*OSML (basic), showed a high homology with tobacco osmotin [15]. As have been shown that the expression of tobacco osmotin enhances salinity tolerance in transgenic soybean [14], our hypothesis was that drought tolerance could be achieved in soybean plants transformed with *Sn*OLP due to crosstalk between these two abiotic stresses [13]. Thus, the objective of the present study was to investigate the usefulness of the *Sn*OLP protein in developing transgenic soybean with enhanced drought tolerance. This is the first study to report that the *SnOLP* gene under the control of the UBQ3 promoter (UBQ3-P) from *A. thaliana* was transferred into the soybean genome. Our results showed an improvement of physiological responses when transgenic plants were subjected to water deficit.

## Methods

### Plant transformation

**Figure 1:**
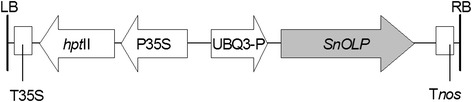
**Diagram of the T-DNA region of the binary vector pCL1390-UBQ3-** ***Sn*** **OLP.** LB: T-DNA left border, RB: right border, T35S: Cauliflower mosaic virus (CaMV) 35S terminator, *hpt*II: hygromycin phosphotransferase gene, P35S: CaMV 35S promoter, UBQ3-P: Ubiquitin 3 promoter from *A. thaliana*, *Sn*OLP: *Solanum nigrum* osmotin-encoding sequence, T*nos*: nopaline synthase gene terminator.

Embryogenic tissues induced from immature cotyledons of soybean cultivar Bragg were transformed by particle bombardment as described by Droste [16]. The pCL1390-UBQ3-*Sn*OLP vector, a pCAMBIA1390 derivative (Cambia.org), contains the complete *SnOLP* gene ORF (GenBank accession AF450276) driven by the UBQ3 promoter (UBQ3-P) from *A. thaliana* and the hygromycin-phosphotransferase marker gene (*hpt* II) driven by the cauliflower mosaic virus (CaMV) 35S promoter (Figure 1).

Selection of hygromycin resistant embryogenic tissues, embryo histodifferentiation, conversion into plants and acclimation were carried out using the methodology previously described [16,17].

Hygromycin-resistant embryogenic tissues were visually selected and separately cultured for establishment and proliferation of lines corresponding to putative independent transformation events. Thus, plants were recovered from three putative transformation events: B1, B2 and B3 lines. Plants derived from an independent piece of hygromycin-resistant tissue were noted as being clone plants.

Plants derived from non-transformed embryogenic tissues subjected to the same culture conditions were recovered and used as controls for molecular characterization.

### PCR analysis

Genomic DNA was extracted from all recovered putative transformed plants (B1, B2 and B3 lines) and from untransformed soybean plants using the CTAB method described by Doyle and Doyle [18]. PCR analyses were performed using specific primers for the *SnOLP* gene (PPS1-foward 5’-CGCGGATCCATGGGCTACTTGAGATCT-3’ and PCPT-reverse 5’-CCCAAGCTTTTACTTGGCCACTTCATC-3’ [15]), which amplify a 744-bp DNA fragment, and for the *hpt*II gene (forward 5’-GCGATTGCTGATCCCCATGTGTAT-3’ and reverse 5’-GGTTTCCACTATCGGCGAGTACTT-3’), which amplify a 512-bp DNA fragment. The PCR reaction mixture consisted of 100 ng of template DNA, 0.2 mM dNTPs, 1.5 mM MgCl_2_, 1X Taq Buffer, 2 units of Taq® DNA Polymerase (Invitrogen), and 0.5 μM of each primer. Reactions were hot-started (3 min at 94°C) and subjected to 25 cycles as follows: 1 min at 94°C, 1 min at 50°C and 2 min at 72°C with a final extension of 72°C for 5 min. All amplification reactions were carried out in a PCR Express Thermal Cycler (Thermo Hybaid, UK). PCR-amplified products were analyzed in 1% agarose gel, stained with ethidium bromide and visualized under UV light.

### Protein expression analysis

For protein expression analysis, 0.2 g of fresh leaf tissue was excised from transgenic T_0_ plants and non-transgenic plants and homogenized in 500 μL of extraction buffer containing 50 mM of 1 M Tris–HCl (pH 6.8), 0.2% (w/v) polyvinylpyrrolidone (PVP-40) and 1% (v/v) β-mercaptoethanol. Samples were stirred for 30 min at 4°C and then clarified by centrifugation at 10,000 g. The protein content in the crude extract was determined by the Bradford method [19], using bovine serum albumin as standard. For each plant, approximately 50 μg of crude protein extract was subjected to 12% (w/v) sodium dodecyl sulfate-polyacrylamide gel electrophoresis (SDS-PAGE) and transferred to a nitrocellulose membrane. The presence of the *Sn*OLP protein was detected using polyclonal antibody specific for tobacco osmotin (kindly supplied by Dr. Bernard Fritig and Dr. Pierrette Geoffroy, Institut de Biologie Moléculaire des Plantes du C.N.R.S, France). The protein bands were visualized using the ECL *Western Blot* Detection and Analysis System (GE Healthcare). To disrupt less-specific interaction more stringent conditions were used by including detergent (0.1% Tween-20) in the wash solution.

### Progeny analysis

T_1_ seeds obtained from self-fertilization of two T_0_ plants (one representative from B1 line and one from B3) were sown in pots containing soil and maintained in greenhouse. All T_1_ plants were screened for the presence of the *SnOLP* and *hpt*II genes by PCR. Subsequent generations were obtained by self-fertilization of transgenic plants. Homozygous plants were detected in T_3_ generation by progeny tests and confirmed by PCR. Homozygous transgenic condition was monitored up to T_7_.

T_5_ homozygous transgenic plants were crossed, as pollen donors, with non-transgenic plants of BRS Fepagro 24 and BRS 211 soybean cultivars. *SnOLP* positive F_1_ plants obtained from crosses were self-fertilized to produce the F_2_ generation. F_2_ plants were screened for the presence of the *SnOLP* gene.

### Transgene copy number estimation by quantitative Real Time PCR (qPCR)

One T_5_ homozygous plant from each B1 and B3 transgenic lines was assayed. Transgene copy number was estimated using relative quantification by qPCR standard curve analysis [20]. The curve was determined by the quantification of an endogenous gene in different DNA dilutions (1:100, 1:1,000 and 1:10,000). Lectin was chosen as the endogenous gene. Two lectin-encoding genes are present in soybean genome, this means there are four alleles in the homozygous diploid genome. The dilution 1:10,000 was hypothetically supposed to contain 4 alleles, the 1:1,000 40 alleles and 1:100 400 alleles. The copy number of transgenes in the same DNA dilutions was automatically calculated in proportion to that of the endogenous lectin genes using the StepOne Applied Biosystem Real-time Cycler™ (Quantification – standard curve experiment).

Primer pairs with a Tm at 60°C were designed to amplify gene sequences corresponding to *Sn*OLP (forward 5’-CAACTTCGATGGTGCTGGTA-3’ and reverse 5’-TCA AAG CGT ATT CGG CTA GG-3’), *hpt*II (forward 5’-TGGTTGGCTTGTATGGAGCAGCAG-3’ and reverse 5’-TGGTCAAGACCAATGCGGAGCATA-3’) and a lectin gene (forward 5’-TACCTATGATGCCTCCACCA-3’ and reverse 5’-GAGAACCCTATCCTCACCCA-3’).

qPCR was carried out under the following cycling conditions: 5 min at 94°C; followed by 40 repetitions of 10 s at 94°C, 15 s at 60°C and 15 s at 72°C; and 2 min at 40°C. A melting curve analysis was performed at the end of the PCR run, over the range 55-99°C, increasing the temperature stepwise by 0.1°C every 1 s. Each 25-μL reaction contained 12.5 μL diluted DNA template, 1x PCR buffer (Invitrogen, São Paulo, Brazil), 2.4 mM MgCl_2_, 0.024 mM dNTP, 0.1 μM each primer, 2.5 μL SYBR-Green (1:100.000, Molecular Probes Inc., Eugene, USA) and 0.3 U Platinum Taq DNA Polymerase (Invitrogen, São Paulo, Brazil). PCRs were performed in technical quadruplicates, and no-template reactions were used as negative controls.

### Plant growth, drought treatment and physiological analysis

A preliminary test was performed to analyze the behavior of transgenic soybean plants under drought conditions. Eight non-transgenic Bragg plants and eight T_6_ homozygous plants from each transgenic line (B1 and B3) were grown in 1-L plastic pots for 26 days in greenhouse. Plants were assessed for tolerance to water deficit stress by withholding irrigation for 10 days. Plants were monitored daily for wilting.

A second experiment was carried out to provide detailed characterization of physiological parameters in T_7_ transgenic plants subjected to drought stress. Plants were individually grown in PVC columns (100 cm in height and 35 cm in diameter) filled with turf and vermiculite (1:1 v/v), natural phosphate and macronutrients (Terral, TrueMix, Brazil). Plants were maintained in greenhouse at 28 ± 2°C and 60 ± 10% relative air humidity. Photosynthetically active radiation (PAR) was measured using a Quantum Sensor LI-COR (Q-45556) attached to a LI-COR 6400 (LICOR-6400, LI-COR Inc., Lincoln, NE, USA). The photosynthetic photon flux density (PPFD) varied from 647 to 1020 μmol m^−2^ s^−1^. The experiment was carried out with a completely randomized design using the two transgenic lines (B1 and B3) and the Bragg wild-type (WT) plants, under two water regimes (watered/always irrigated and stressed/with a water deficit imposed at beginning of pod formation – R_3_ stage) with five biological replicates. The experimental unit was one soybean plant grown in a PVC column.

Before sowing, the substrate was dried at 105°C. Each PVC column was filled with 43.0 kg of substrate. Six small holes were made in the column bottom to facilitate initial drainage. Subsequently, columns were irrigated with water up to saturation and covered with plastic bags, and excess water was allowed to drain out for 24 hours. Then, drainage holes were sealed, and columns were weighed for field capacity determination. Three seeds were sown per column, leaving one plant per column after thinning on the day 12 after sowing. Every two weeks, 0.5 L of half-strength “Hoagland” solution [21] was applied to each column. The plants were irrigated regularly with water to maintain the substrate at field capacity up to the R_3_ stage (46 days after emergence; beginning of pod formation). After that, plants were separated into two groups: one continued to receive regular irrigation (watered plants), and the other was subjected to water deficit (stressed plants).

Measurements of water potential were performed as described [22] using an Oregon Corvallis pressure chamber, 97330 (PMS Instrument Company, Albany, OR, USA). Leaves were collected from the upper portion of the middle third of each plant.

The net assimilation rate (*P*n), stomatal conductance (*gs*), and the transpiration rate (*E*) were measured from 09:00 to 11:00 a.m. under artificial, saturating photosynthetic photon flux (PPF) (900 μmol m^−2^ s^−1^), using a portable photosynthesis system infrared gas analyzer (Li-cor 6400XTR, Nebraska, USA.). Measurements were recorded at one, six and twelve days after imposing water stress.

At harvest maturity, 100-grain weight and grain production per plant were determined in five plants of each transgenic line (B1 and B3) and five WT plants.

### Statistical analyses

The segregation rates of the T_1_ progenies of transgenic soybean plants, as well as the segregation ratios in the F_2_ generation from crosses between T_5_ homozygous transgenic plants and non-transgenic plants of two commercial cultivars, were analyzed using the chi-square test to confirm the expected Mendelian segregation pattern of 3:1 (transgenic:non-transgenic plants).

Predawn leaf water potential, net CO_2_ assimilation rate, stomatal conductance, transpiration rate, grain production per plant and 100-grain weight of the transgenic plants grown under two water regimes were compared to those of non-transgenic ones grown in the same environmental conditions. Data were subjected to analysis of variance (ANOVA) and comparison of means was performed with Student’s *t*-test using SPSS Statistics software. Physiological parameters obtained for B1 and B3 lines were compared to those of WT-plants for each evaluation day and water regime.

## Results and discussion

Twelve plants were recovered from three independent pieces of hygromycin-resistant embryogenic clusters. The three putative independent transformed lines were named B1, B2 and B3. Ten clonal plants derived from B1, and one plant derived from each of the B2 and B3 lines. The recovered plants reached maturity, flowered and set seeds.

**Figure 2:**
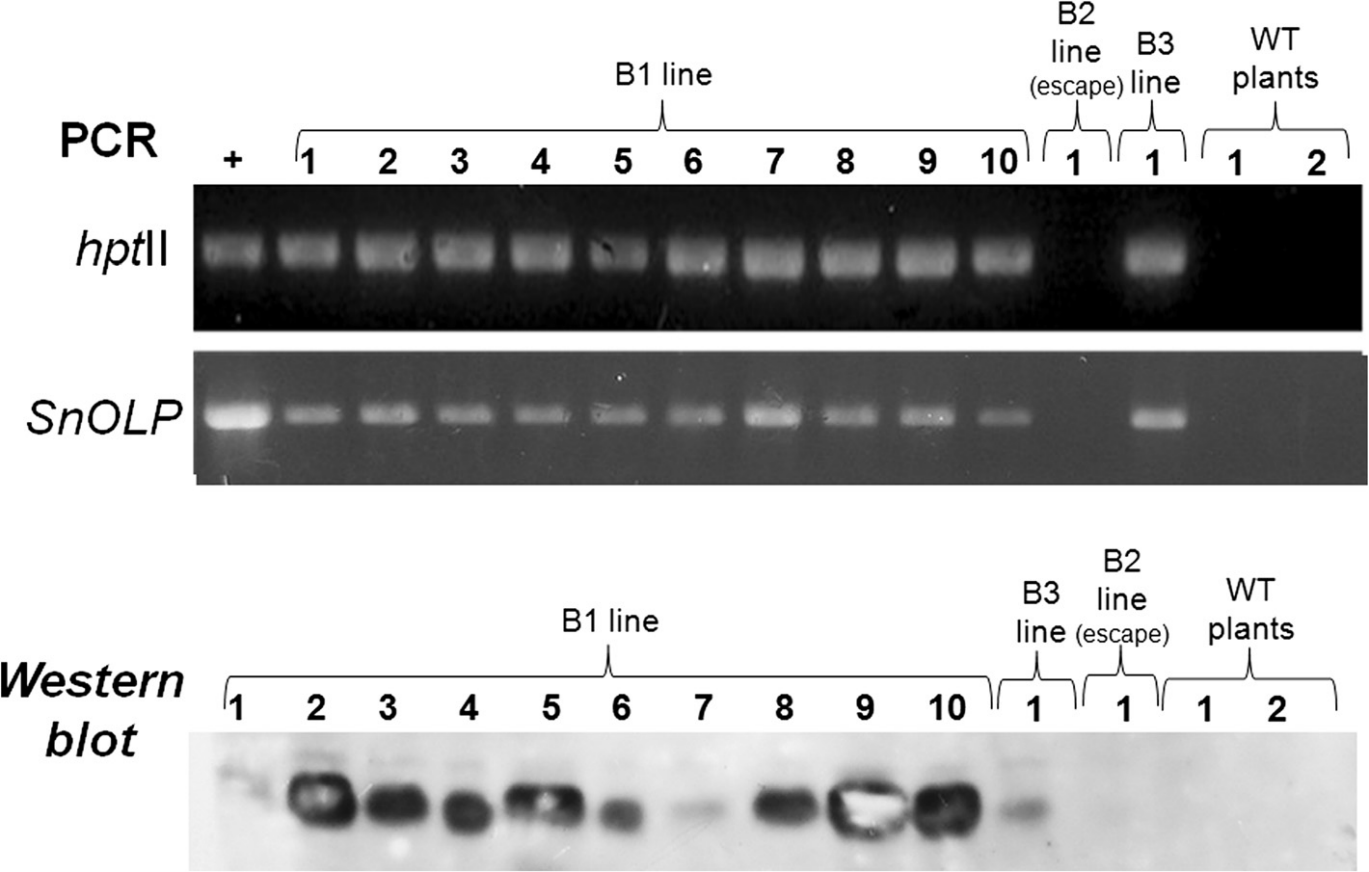
**Molecular analysis in transgenic soybean plants.** PCR products of *hpt*II and *SnOLP* genes amplified from DNA extracted from transgenic plants and controls. Detection of the *Sn*OLP protein (~27 kDa) in soybean plants by *Western blot* hybridization. +: vector pCL1390-UBQ3-*Sn*OLP (PCR positive control). B1, B2 and B3 lines: plants recovered from three putative transformation events. B1 line was represented by 10 clone plants. B2 and B3 lines were represented by one plant each. Two Bragg wild-type (WT) plants were used as negative controls.

The stable integration of *SnOLP* and *hpt*II transgenes into the genomes of hygromycin-resistant soybean plants was confirmed by PCR. Fragments with the expected size (512 bp for *hpt*II and 744 bp for *SnOLP*) were detected in all plants from B1 as well as the plant from B3 lines (Figure 2). No PCR product for the tested gene was observed for the plant from B2 line, which was considered an “escape”. The expression of *SnOLP* was confirmed by Western *blot*. Using a polyclonal antibody against tobacco osmotin, a single band of approximately 27 kDa, corresponding to the *Sn*OLP protein, was detected only in PCR-positive plants from B1 and B3 lines (Figure 2). No band was observed in non-transformed plants. Altogether, these data confirmed the successful production of soybean transgenic plants expressing the *SnOLP* gene.

T_1_ progenies were used for transgene segregation analysis. Seeds harvested from one T_0_ plant representative of B1 and from the T_0_ plant from B3 lines were sowed, and T_1_ generation was analyzed by PCR. Both progenies segregated 3:1 for *hpt*II and *SnOLP* transgenes (*p* > 0.05), indicating single locus insertions (Table 1). The transgene locus is considered to be hemizygous in the primary T_0_ transformant. Therefore, transgenes are generally expected to behave as dominant single genes and segregate in a 3:1 ratio when the plant is self-pollinated.

**Table 1:** **Segregation rates of the T**_**1**_ **progeny of two T**_**0**_ **transgenic soybean plants (one from B1 line and one from B3)** ^(*)^

**Line**	**T**_**0**_ **Plants**	**Number of plants**			**Expected ratio**	***p*** **value**
		**T**_**1**_	***hpt*** **II/** ***Sn*** **OLP +**	***hpt*** **II/** ***Sn*** **OLP -**		
B1	10	30	24	6	3:1	>0.05
B3	1	20	11	9	3:1	>0.05

^(*)^Segregation ratios were tested using the chi-square test.

Homozygous plants were identified in the T_3_ generation. B1 and B3 homozygous transgenic lines were propagated up to T_7_ without the loss of transgenes. As predicted, crosses between homozygous transgenic plants and non-transformed plants of two commercial cultivars (Fepagro 24 and BRS 211) produced only *hpt*II/*SnOLP*-positive F_1_ plants. The 3 transgenic:1 non-transgenic ratio (*p* > 0.05) observed in the F_2_ generation confirmed the Mendelian pattern for a single locus (Table 2).

**Table 2:** **Segregation ratios in the F**_**2**_ **generation from crosses between T**_**5**_ **homozygous transgenic plants (B1 and B3 lines) and non-transgenic plants (Fepagro 24 and BRS 211 cultivars)** ^(*)^

**Cross**	**Number of plants**		**Expected ratio**	***p*** **value**
	***hpt*** **II/** ***Sn*** **OLP +**	***hpt*** **II/** ***Sn*** **OLP -**		
Fepagro 24 X B1 line	25	5	3:1	>0.05
BRS 211 X B1 line	20	10	3:1	>0.05
Fepagro 24 X B3 line	21	9	3:1	>0.05
BRS11 X B3 line	26	4	3:1	>0.05

^(*)^Segregation ratios were tested using the chi-square test.

The copy number of transgenes was calculated by qPCR proportionally to the endogenous lectin genes (Table 3). A single insertion of *SnOLP* was present in both the B1 and B3 lines. Differences between lines were detected for the *hpt*II gene, i.e., one copy was inserted in B1 and two in B3. This result confirms that the transformed lines correspond to two independent transformation events. Transgenes rearrangements following particle bombardment have been widely reported [23,24]. The insertion of one or a few transgene copies into the plant genome by particle bombardment is rare but has been previously reported in soybean [25].

**Table 3:** **Number of recombinant** ***Sn*** **OLP and** ***hpt*** **II copies integrated into the transgenic plant genome**

**Transformation line**	**Number of copies**	
	***Sn*** **OLP**	***hpt*** **II**
B1	1	1
B3	1	2

Estimative was performed by qPCR comparing DNA quantification of the transgene and a reference gene (lectin gene), with known copy number.

Transgenic plants with a strong constitutive expression of functional genes often suffer from undesirable phenotypes including growth retardation, abnormal development, and reduced seed production. The plants obtained in the present study, in which the transgene is controlled by the constitutive UBQ3 promoter, did not show any remarkable phenotypic alteration on the course of the seven analyzed generations under regular watering regime.

**Figure 3:**
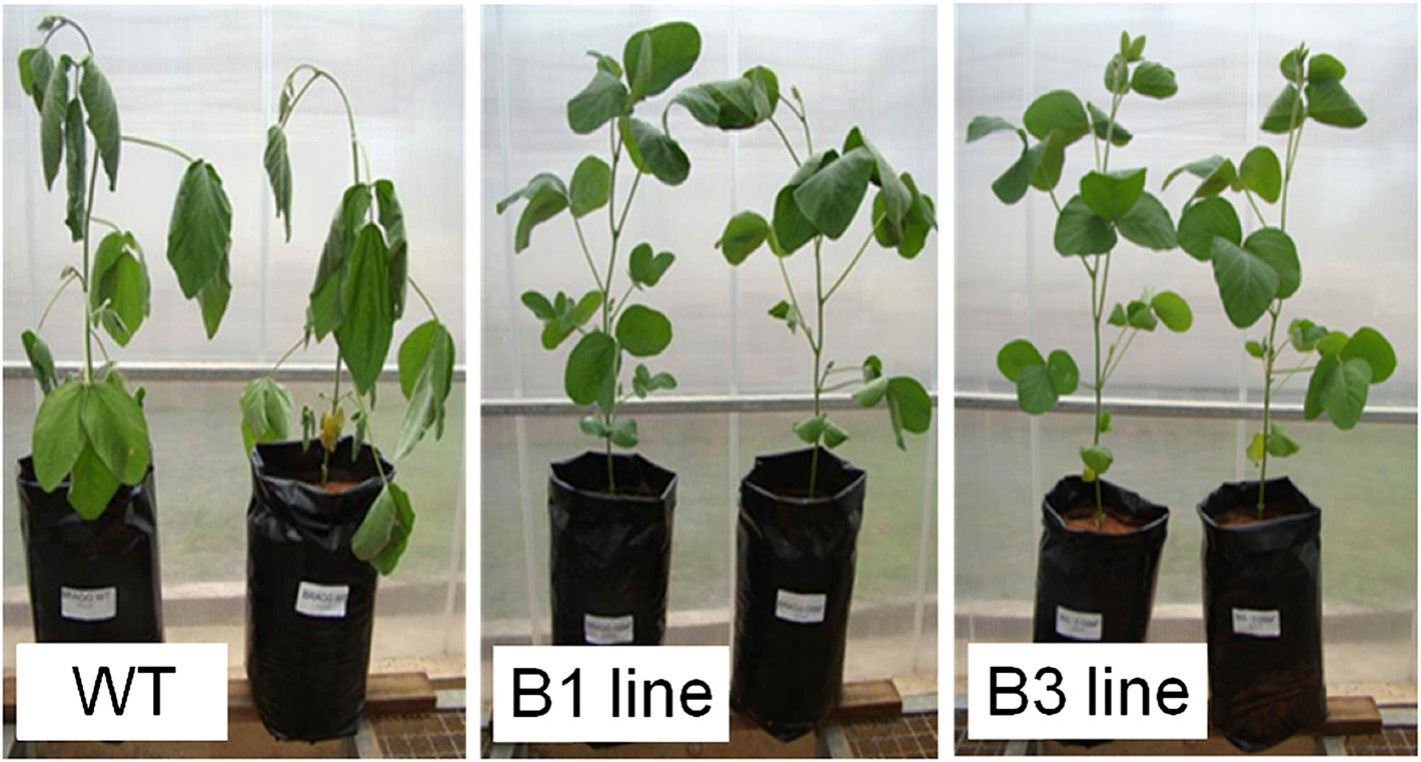
**Plants under water deficit.** Non-transgenic (WT) and transgenic plants expressing *Sn*OLP after withholding water for seven days. Eight non-transgenic Bragg plants and eight T_5_ transgenic homozygous plants from each transgenic line (B1 and B3) were grown in 1-L plastic pots for 26 days in a greenhouse. Plants were assessed for tolerance to water deficit stress by withholding irrigation and were monitored daily for wilting.

Differences were observed between transgenic and control plants subjected to water deficit. The results of the preliminary test showed that transgenic plants of both lines (B1 and B3) performed better than the control ones. Results obtained from two out of eight plants/line are shown in Figure 3. After seven days without irrigation, non-transformed plants exhibited visible loss of turgidity, becoming limp and droopy. Transgenic plants appeared quite healthy up to 10 days without irrigation, when they started wilting (data not shown). Similarly, transgenic cotton and tomato plants overexpressing tobacco osmotin showed less severe wilting compared to controls [12,13].

**Figure 4:**
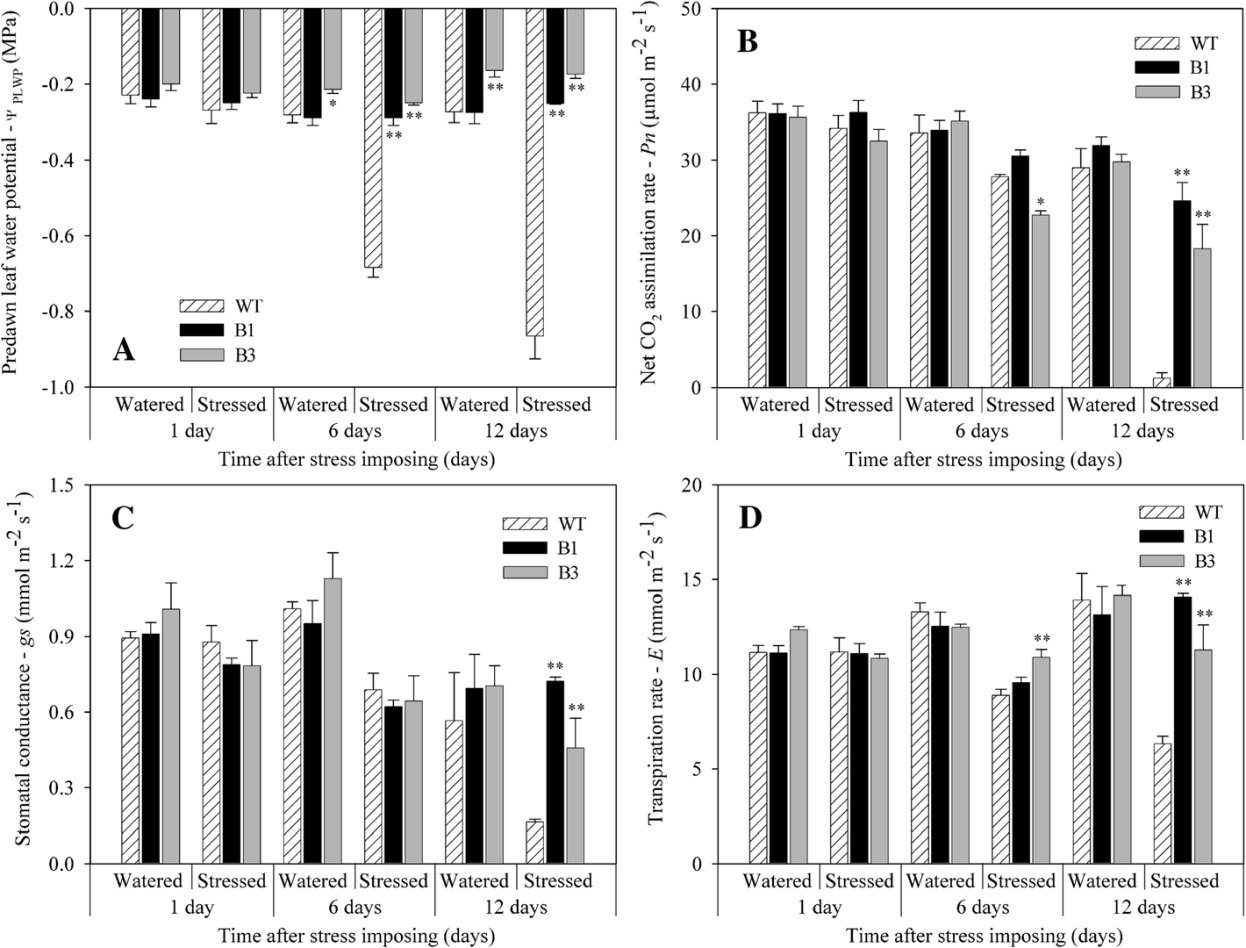
**Physiological evaluation of transgenic plants under water deficit.** Predawn leaf water potential **(A)**, net CO_2_ assimilation rate **(B)**, stomatal conductance **(C)** and transpiration rate **(D)** of transgenic B1 and B3 lines and wild-type (WT) plants grown under two water regimes. Water deficit was imposed at R_3_ stage (beginning of pod formation). Measurements were recorded at one, six and twelve days after imposing water stress. Asterisks indicate significant differences of B1 or B3 lines compared to wild-type plants in each evaluation day and water regime (Student’s *t*-test, *: p < 0.05, **: p < 0.01). Values are means ± SD (n = 5).

In the second experiment, physiological parameters were evaluated under well-watered and drought stress conditions. The predawn leaf water potential (Ψ_PLWP_) has been used as a tool to assess plant water status [26] because it integrates the effects of several drought-adaptive traits [27]. In our study, no difference was observed between transgenic and non-transgenic plants under regular irrigation (p > 0.05). Under water deficit, Ψ_PLWP_ was significantly lower in non-transgenic plants when compared to transgenic ones. The difference between transgenic and non-transgenic plants became evident after 6 days of water shortage (p < 0.01) (Figure 4A). This result indicates that plants overexpressing *SnOLP* are protected against dehydration.

It has been shown that gas exchange variables in soybean are reduced by drought. Water deficit causes a decrease in photosynthetic rate by means of decreased stomatal conductance to carbon dioxide (CO_2_) as well as photosynthetic metabolic potential [28]. Under watered condition, net CO_2_ assimilation rate (*P*n) was not significantly different in transgenic plants when compared to non-transgenic plants (p > 0.05) (Figure 4B). *P*n decreased in all soybean plants under drought stress. However, *P*n in transgenic plants was significantly higher than in wild-type plants at 12-day-drought stress (p < 0.01) (Figure 4B). This result indicates that soybean plants overexpressing *SnOLP* continue to assimilate CO_2_ even under drought stress.

The reduction in the photosynthetic rate is usually due to low stomatal conductance (*g*s), which reduces transpiration rate and internal CO_2_ concentration, leading to a decrease in plant development [29] and inhibition of photosynthesis [30]. Stomatal conductance (*g*s) was not significantly different in transgenic plants when compared to WT plants under watered conditions (Figure 4C). The *g*s values of all plants decreased during the drought period, but transgenic plants had significantly greater *gs* than those of the WT plants at 12 day-drought stress (p < 0.01). Plants with higher *g*s are desirable in soybean breeding strategies because low *gs* is related to decreased productivity [31].

Similarly, transpiration rate (*E*) did not differ in well-watered WT and transgenic plants (p > 0.05) (Figure 4D). During exposure to drought, transpiration increased in transgenic plants compared to WT ones. The transgenic plants of both lines showed significant differences in *E* (p < 0.01) starting on days 6 and 12 after stress was imposed.

**Figure 5:**
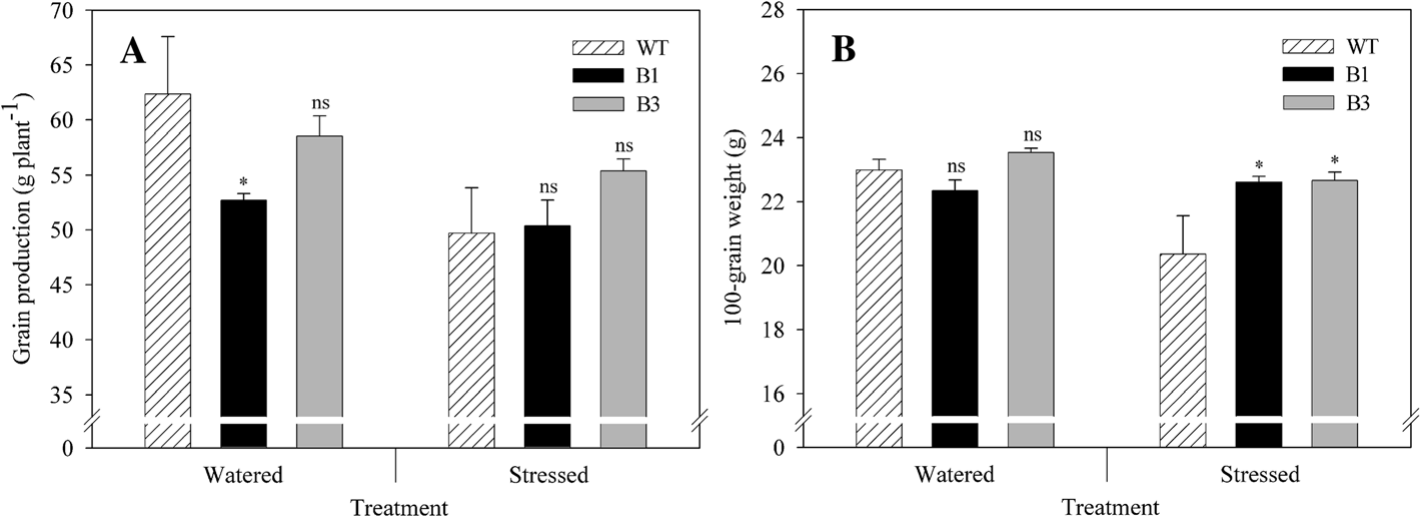
**Productivity of transgenic plants submitted to water deficit.** Grain production per plant **(A)** and 100-grain weight **(B)** of transgenic B1 and B3 lines and wild-type (WT) plants grown under two water regimes. Water deficit was imposed for 12 days at R3 stage (beginning of pod formation). Asterisks indicate significant differences of B1 or B3 lines compared to wild-type plants in each water regime (Student’s t-test, p < 0.05). ns = non-significant difference. Values are means ± SD (n = 5).

Grain production and 100-grain weight were affected by water supply (Figure 5). For WT plants, grain productivity decreased approximately 20% when water shortage was imposed (Figure 5A). However, the reduction in grain production under drought stress for transgenic plants varied between 4.5% and 5.3% for B1 and B3 lines, respectively. The decrease in grain yield observed under water stress compared to watered conditions most likely occurred due to the loss of legumes since water stress was imposed at beginning of pod formation (R_3_ stage). Therefore, the reduction was more pronounced for WT plants than for transgenic ones, showing that transgenic plants were able to maintain higher growth rates compared to WT plants during water shortage.

Under watered conditions, grain production for the B1 line was significantly lower compared to WT plants (p < 0.05) (Figure 5A). In fact, there are reports demonstrating that transgenic plants carrying genes coding for osmoprotectants under a constitutive promoter may present morphological changes as well as growth rate and seed grain yield reduction [32,33]. However, there is no sufficient basis to assume a negative association between osmotic adjustment and grain yield potential, especially considering the study carried out by Richardson and McCree [34], which demonstrated that the metabolic cost of storing photosynthate and using it for osmotic adjustment in sorghum was lower than the cost of converting it into shoot biomass.

Under watered conditions, 100-grain weight was similar for WT and transgenic plants (B1 and B3 lines) (p > 0.05) (Figure 5B). However, under water stress, 100-grain weight decreased approximately 12% for WT plants, while approximately 1.1% (B1) and 3.7% (B3) for transgenic plants. Moreover, plants from B1 and B3 lines exposed to water shortage showed higher 100-grain weights than WT plants (p < 0.05). The higher 100-grain weight observed for transgenic plants compared to WT plants may be explained by the effect of drought stress on net CO_2_ assimilation rate (*P*n). As shown in Figure 4B, *P*n in transgenic plants was significantly higher than in WT plants under water shortage. As discussed above higher CO_2_ assimilation increases grain-filling rate and duration as well as grain weight (Figure 5B).

Altogether our results indicate that transgenic soybean plants expressing *Sn*OLP can sustain higher net CO_2_ assimilation, stomatal conductance, and transpiration compared to non-transformed plants. These data suggest that transgenic plants were able to better use internal carbon dioxide. Similar results were observed in soybean plants expressing an osmotin from tobacco when subjected to salinity stress [14].

## Conclusions

The usefulness of the *Sn*OLP protein in developing soybean transgenic plants with enhanced tolerance to drought has been demonstrated in this study. This biotechnological strategy combined with conventional genetic breeding approach should contribute to overcome the serious constraint – drought stress – for soybean production.

### Availability of supporting data

*Solanum nigrum* osmotin-like protein precursor (OLP) gene, complete CDS (GenBank AF450276) - http://www.ncbi.nlm.nih.gov/nuccore/AF450276.
